# Effect of Everolimus versus Bone Marrow-Derived Stem Cells on Glomerular Injury in a Rat Model of Glomerulonephritis: A Preventive, Predictive and Personalized Implication

**DOI:** 10.3390/ijms23010344

**Published:** 2021-12-29

**Authors:** Mohamed M. Zedan, Ahmed K. Mansour, Ashraf A. Bakr, Mohamed A. Sobh, Hesam Khodadadi, Evila Lopes Salles, Abdulmohsin Alhashim, Babak Baban, Olga Golubnitschaja, Ahmed A. Elmarakby

**Affiliations:** 1 Department of Pediatric, Faculty of Medicine, Mansoura University, Mansoura 35516, Egypt; mzedan@mans.edu (M.M.Z.); amansour@mansoura.edu (A.K.M.); Abakr@mans.edu (A.A.B.); 2 Urology and Nephrology Center, Faculty of Medicine, Mansoura University, Mansoura 35516, Egypt; msobh@mansoura.edu; 3 Department of Oral Biology & Diagnostic Sciences, Augusta University, Augusta, GA 30912, USA; HCHAMGORDANI@augusta.edu (H.K.); esalles@augusta.edu (E.L.S.); bbaban@augusta.edu (B.B.); 4 Department of General Dentistry, Augusta University, Augusta, GA 30912, USA; aalhashim@augusta.edu; 5 Predictive, Preventive and Personalised (3P) Medicine, Department of Radiation Oncology, University Hospital Bonn, Rheinische Friedrich-Wilhelms-Universität Bonn, 53127 Bonn, Germany; 6 Department of Pharmacology & Toxicology, Faculty of Pharmacy, Mansoura University, Mansoura 35516, Egypt

**Keywords:** everolimus, anti-Thy1, BMDSCs, glomerular injury, renal inflammation, apoptosis

## Abstract

Glomerular endothelial injury and effectiveness of glomerular endothelial repair play a crucial role in the progression of glomerulonephritis. Although the potent immune suppressive everolimus is increasingly used in renal transplant patients, adverse effects of its chronic use have been reported clinically in human glomerulonephritis and experimental renal disease. Recent studies suggest that progenitor stem cells could enhance glomerular endothelial repair with minimal adverse effects. Increasing evidence supports the notion that stem cell therapy and regenerative medicine can be effectively used in pathological conditions within the predictive, preventive and personalized medicine (PPPM) paradigm. In this study, using an experimental model of glomerulonephritis, we tested whether bone marrow-derived stem cells (BMDSCs) could provide better effect over everolimus in attenuating glomerular injury and improving the repair process in a rat model of glomerulonephritis. Anti-Thy1 glomerulonephritis was induced in male Sprague Dawley rats by injection of an antibody against Thy1, which is mainly expressed on glomerular mesangial cells. Additional groups of rats were treated with the immunosuppressant everolimus daily after the injection of anti-Thy1 or injected with single bolus dose of BMDSCs after one week of injection of anti-Thy1 (n = 6–8). Nine days after injection of anti-Thy1, glomerular albumin permeability and albuminuria were significantly increased when compared to control group (*p* < 0.05). Compared to BMDSCs, everolimus was significantly effective in attenuating glomerular injury, nephrinuria and podocalyxin excretion levels as well as in reducing inflammatory responses and apoptosis. Our findings suggest that bolus injection of BMDSCs fails to improve glomerular injury whereas everolimus slows the progression of glomerular injury in Anti-Thy-1 induced glomerulonephritis. Thus, everolimus could be used at the early stage of glomerulonephritis, suggesting potential implications of PPPM in the treatment of progressive renal injury.

## 1. Introduction

The physiological role of the kidney in the regulation of fluid homeostasis and blood pressure is well characterized [1]. Glomerulonephritis is a pathological kidney disease in which glomeruli are inflamed and damaged and fail to effectively remove waste and fluid [2]. The disease is mainly characterized by elevation in blood pressure, hematuria, proteinuria and anemia [2]. The incidence and progression of glomerulonephritis are mainly influenced by the degree of glomerular endothelial injury and the effectiveness of glomerular endothelial repair [3]. Considerable research efforts have recently been undertaken to examine new candidates for glomerular endothelial repair. Anti-Thy1 nephritis is an experimental model of immune-mediated glomerular disease, induced by an antibody directed against the Thy1 antigen (CD90) which is constitutively expressed in mesangial cells [4]. Anti-Thy1 nephritis is characterized by acute mesangiolysis followed by early inflammatory cell infiltration, repopulation of the mesangium by new mesangial cells and accumulation of mesangial matrix [4]. This model is used experimentally to test new drug candidates for their potential use to halt the progression of glomerulonephritis [5].

Infiltration of immune cells plays a critical role in the pathogenesis of renal injury and its progression to end-stage renal disease [6]. Immunosuppressive treatment has been shown to halt the progression of renal injury in an experimental model of glomerulonephritis [7]. In organ transplantation, calcineurin inhibitors are still used in many immunosuppressive protocols; however, they are known to induce nephrotoxicity [8]. Recently, the novel mammalian target of rapamycin (mTOR) inhibitor sirolimus or its derivative everolimus is increasingly used in renal transplant patients because of the potent immunosuppressive properties with a lesser degree of nephrotoxicity [9]. Nevertheless, adverse effects of using mTOR inhibitors have been reported clinically in human renal allografts, glomerulonephritis and experimental renal disease [10,11,12,13]. Although everolimus was shown to disrupt the repair process in the anti-Thy1 nephritis model, another study demonstrated that it did not disturb the long-term repair process in a rat model of renal thrombotic microangiopathy [14]. In the remnant kidney model, everolimus worsened the progression of renal injury via increasing proteinuria, glomerulosclerosis, interstitial fibrosis and glomerular inflammation and decreasing creatinine clearance [15]. Thus, short-term vs. long-term use of everolimus in halting the progression of renal injury still requires further investigation.

Bone marrow-derived stem cells (BMDSCs) contribute to cell repair in various tissue types, including the kidneys [4,16]. Besides their promising role in regenerating injured organs in experimental and human disease [17,18], BMDSCs appear to have a distinct immunomodulatory action that results in a protective effect in immune-mediated diseases. For example, bone marrow cells have been shown to differentiate into endothelial or mesangial cells in anti-Thy1-induced nephritis in rats [17,18,19]. However, previous studies also indicate that mesenchymal stromal cells failed to replace injured cells in anti-Thy1 nephritis but improved anti-Thy1 nephritis via preventing cytokine-driven inflammation [4].

Given the conflicting data on the safety and efficacy of everolimus in halting the progression of glomerulonephritis and the promising role of BMDSCs in the repair of injured tissues with minimal adverse effects, the current study was designed to compare the protective effect of everolimus vs. BMDSCs on anti-Thy1-induced glomerulonephritis in rats. Our study investigated if everolimus or BMDSC treatment ameliorates experimental glomerulonephritis by restoring glomerular function through reducing apoptosis and/or preventing cytokine-induced inflammation. Because personalized medicine plays a central role in establishing a protective platform in the treatment of cardio-renal diseases, the outcomes of the current study would be an essential tool in improving our understanding of renal injuries, magnifying the significance of PPPM in the prediction of disease progression.

## 2. Results

**Figure 1:**
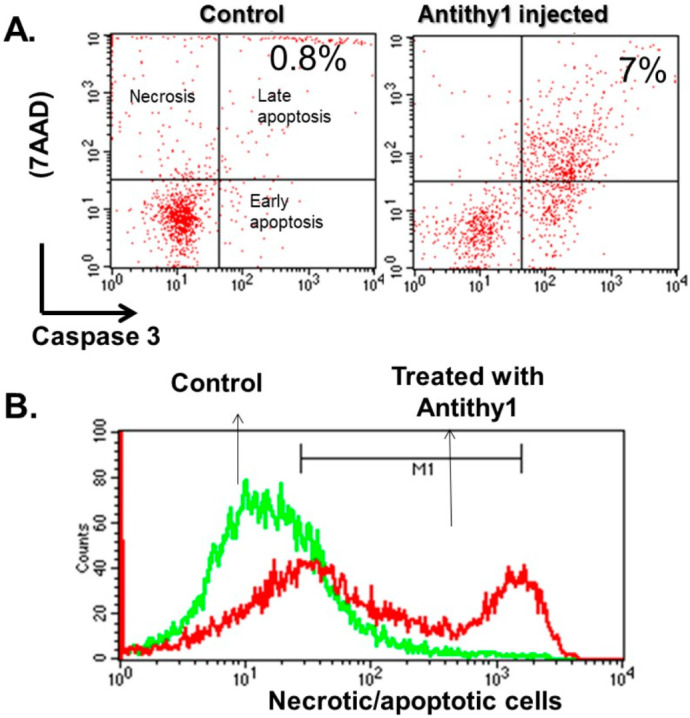
Assessment of apoptosis (caspase 3) and necrosis (7-AAD) in glomeruli isolated from control and anti-Thy1-injected rats (1 mg/kg i.v.). Representative of flow cytometry analysis of apoptosis (caspase 3) and necrosis (7-AAD) in two-dimensional dot plot (**A**) and in histogram (**B**) in a comparative mode based on counted total cells. Injection of anti-Thy1 produced 9-fold increase in glomerular apoptosis vs. control.

**Figure 2:**
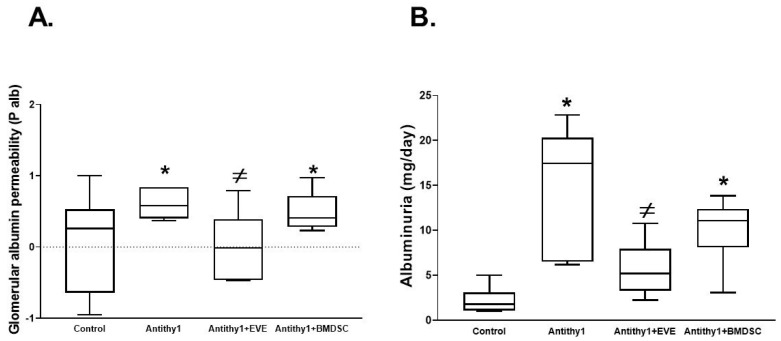
Glomerular permeability to albumin (**A**) and albuminuria (**B**) in control and anti-Thy1-injected rats +/− everolimus (2 mg/kg daily) or single-dose administration of BMDSCs (10^6^ cells/200 µL of PBS, i.v.) after a week of injection of anti-Thy1. Only everolimus treatment significantly reduced the elevation in glomerular permeability to albumin and albuminuria in anti-Thy1-injected rats (n = 6–8, * vs. control and ^≠^ vs. anti-Thy1-injected rats).

**Figure 3:**
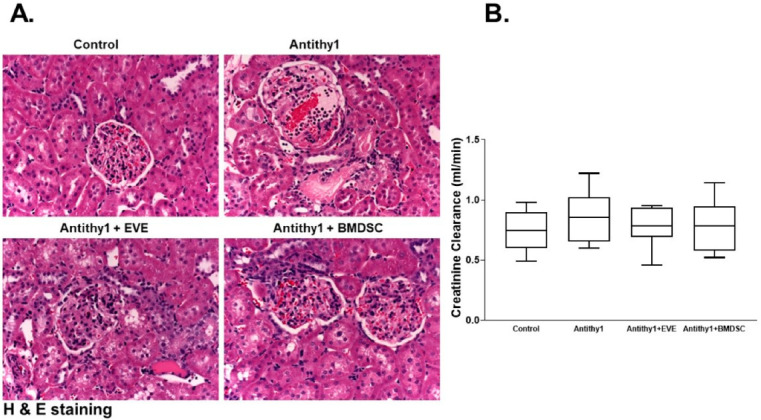
Representative H&E staining images for the kidney section (**A**) and creatinine clearance (**B**) from control and anti-Thy1-injected rats with or without everolimus or BMDSC treatment. Anti-Thy1-injected rats showed hypercellular glomeruli with loss of Bowman space and variable number of lymphocytes. Only everolimus treatment partially restored these pathophysiological changes (n = 4). There was no difference in creatinine clearance among rat groups (n = 6–8).

**Figure 4:**
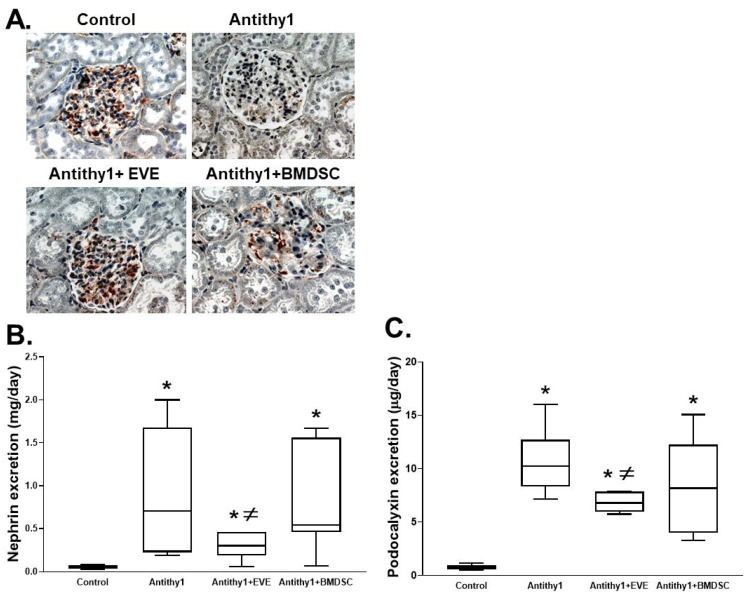
Representative images of immunohistochemical staining of glomerular nephrin expression in kidney sections (**A**), urinary nephrin excretion (**B**) and podocalyxin excretion (**C**) levels in control and anti-Thy-1-injected rats +/− everolimus or BMDSC treatment. Anti-Thy-1 injection markedly increased the loss of glomerular nephrin, nephrinuria and podocalyxin excretion. Only everolimus lowered nephrin and podocalyxin excretion levels as markers of glomerular injury and restored the loss of glomerular nephrin expression in anti-Thy1-injected rats (n = 6–8, * vs. control and ^≠^ vs. anti-Thy1-injected rats).

**Figure 5:**
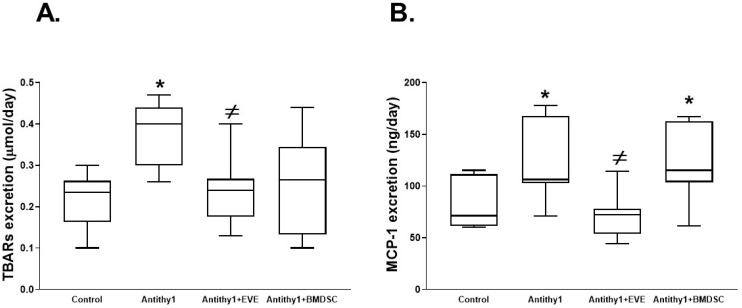
Urinary TBARs excretion (**A**) as a marker of oxidative stress and MCP-1 excretion as a marker of inflammation (**B**) in control and anti-Thy1 rats with or without everolimus or BMDSC treatment. Only everolimus treatment significantly reduced the elevation in urinary TBARs and MCP-1 excretion levels in anti-Thy1-injected rats (n = 6–8, * vs. control and ^≠^ vs. anti-Thy1-injected rats).

We first determined if the injection of anti-Thy1 increased glomerular apoptosis vs. control noninjected rats. Using flow cytometry analysis, injection of anti-Thy1 produced an approximately 9-fold increase in glomerular apoptosis relative to control (Figure 1). We then assessed glomerular albumin permeability and albuminuria as indicative of glomerular injury (Figure 2). Injection of anti-Thy1 significantly increased glomerular albumin permeability (Figure 2A) and albuminuria (Figure 2B). Only everolimus treatment but not BMDSCs significantly reduced the elevation in glomerular permeability to albumin and albuminuria in anti-Thy1-injected rats (Figure 2). Histological analysis of kidney sections using H&E staining also revealed that anti-Thy1 injection produced hypercellular glomeruli with loss of Bowman space and variable number of lymphocytes and these pathological changes were only improved with everolimus treatment (Figure 3A). There was no difference in creatinine clearance as an indirect indication of glomerular filtration rate among all rat groups (Figure 3B). The increase in glomerular injury was further confirmed after anti-Thy1 injection by assessing urinary excretion levels of nephrin and podocalyxin which are glomerular podocyte proteins involved in the proper function of the glomerular filtration barrier. Injection of anti-Thy1 decreased glomerular nephrin expression (Figure 4A) consistent with the elevation in urinary nephrin and podocalyxin excretion levels as markers of glomerular injury (Figure 4B,C). Everolimus treatment prevented the decrease in glomerular nephrin expression (Figure 4A) and significantly decreased nephrin and podocalyxin excretion levels (Figure 4B,C) in anti-Thy1-injected rats, whereas single bolus administration of BMDSCs failed to restore the decrease in glomerular nephrin expression or to improve the markers of glomerular injury. The increase in glomerular injury after anti-Thy1 injection was also associated with a significant elevation in oxidative stress and inflammation as evident by the elevation in urinary TBARs and MCP-1, respectively (Figure 5). Consistent with its glomerular protective effect, everolimus significantly decreased urinary TBARs excretion as a marker of oxidative stress (Figure 5A) and urinary MCP-1 excretion as a marker of inflammation (Figure 5B) in anti-Thy1-injected rats. Administration of BMDSCs failed to lower urinary TBARs or MCP-1 excretion levels after anti-Thy1 injection (Figure 5).

**Figure 6:**
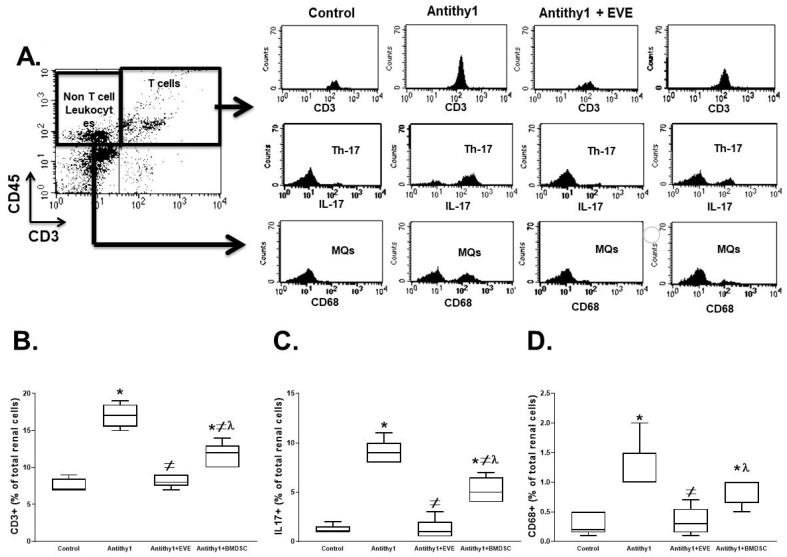
Representative flow cytometry plots (**A**) and % of total renal cells expressing CD3+ (**B**), IL-17+ (**C**) and CD68+ (**D**) as markers of naïve T cells, T helper cells and macrophages, respectively, in control and anti-Thy1-injected rats with or without everolimus or BMDSC treatment. Everolimus treatment prevented the elevation in T cells and IL-17 expression in anti-Thy1-injected rats. Everolimus provided better effect than BMDSCs in preventing the increase in renal macrophage infiltration in anti-Thy1-injected rats (n = 6, * vs. control, ^≠^ vs. anti-Thy1-injected rats and ^λ^ vs. everolimus-treated rats).

**Figure 7:**
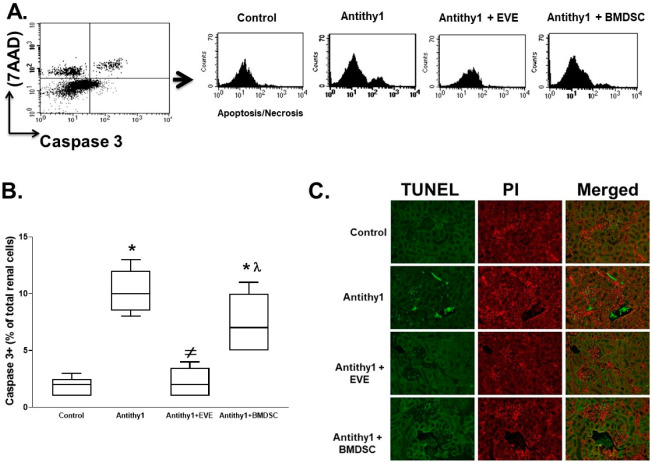
Representative flow cytometry plots (**A**) and % of total renal cells expressing caspase 3+ (**B**) as indicative of apoptosis in control and anti-Thy1-injected rats with or without everolimus or BMDSC treatment. Everolimus was superior to BMDSCs in reducing renal caspase 3 expression in anti-Thy1-injected rats (n = 6, * vs. control, ^≠^ vs. anti-Thy1-injected rats and ^λ^ vs. everolimus-treated rats). The result was further verified using TUNEL assay for renal apoptotic cells in the kidney sections from the same rat group (**C**) where number of renal apoptotic cells increased after anti-Thy1 injection and was only reduced with everolimus treatment.

The elevation in inflammation after anti-Thy1 injection was further confirmed by assessing the expression levels of renal pan T cells, IL-17 and activated macrophages using flow cytometry. As shown in Figure 6, the expression levels of renal pan T cells, IL-17 and activated macrophages (CD68+) were elevated in anti-Thy17 injected rats. Although either everolimus or BMDSC administration reduced the elevation in renal pan T cells, IL-17 and activated macrophages in anti-Thy1-injected rats, everolimus was superior to BMDSCs in reducing renal T cells, IL-17 and activated macrophages (Figure 6). The reduction in expression of renal T cells and macrophages after BDMSC treatment was not necessarily translated to a reduction in renal apoptosis, since BMDSCs failed to reduce the elevation in renal caspase 3 expression in anti-Thy1-injected rats (Figure 7A,B). On the other hand, everolimus significantly attenuated the increase in renal caspase 3 expression in anti-Thy1-injected rats (Figure 7A,B). The ability of everolimus to reduce renal apoptosis was further confirmed using TUNEL assay (Figure 7C). The number of TUNEL positive cells, a marker of apoptosis, in the kidney sections from anti-Thy1-injected rats was higher than that from control rats, and this increase was reduced by everolimus treatment (Figure 7C).

## 3. Discussion

The safety and efficacy of the mTOR inhibitor everolimus in halting the progression of glomerulonephritis require further investigation. On the other hand, BMDSCs show promise for use in the repair of injured tissues with minimal adverse effects. Thus, the aim of the present study was to investigate the reno-protective effects of everolimus vs. BMDSCs on the course of anti-Thy1 nephritis. We utilized anti-Thy1 antibody to target CD90 (Thy1) to induce glomerulonephritis in Sprague Dawley rats, and our findings suggest that daily administration of everolimus to anti-Thy1-injected rats reduced markers of renal injury and glomerular damage. Everolimus also prevented the decrease in glomerular nephrin expression and reduced oxidative stress and inflammation. The reduction in glomerular injury in anti-Thy1-injected rats with everolimus treatment was associated with a reduction in the expression of renal T cells, IL-17 and activated macrophages together with a reduction in renal apoptosis. On the other hand, single bolus administration of BMDSCs one week after induction of glomerulonephritis with anti-Thy1 injection failed to reduce oxidative stress, apoptosis and markers of glomerular injury or to restore glomerular nephrin expression even though BMDSC administration reduced the expression of renal T cells, IL-17 and activated macrophages. These findings suggest that the immunosuppressive effect of the mTOR inhibitor everolimus was effective to reduce renal and glomerular injury via reduction in oxidative stress, inflammation and apoptosis, whereas single-dose administration of BMDSCs was not sufficient to reduce glomerular injury and renal apoptosis.

Anti-Thy1-induced nephritis is an established model of mesangioproliferative glomerulonephritis and is characterized by initial mesangiolysis followed by repair via endothelial and mesangial cell proliferation and accumulation of mesangial matrix [4]. Previous studies have shown that many antiproliferative therapies such as PDGF antagonists, heparin, mycophenolate mofetil and the cell cycle inhibitors provide renal protection in the anti-Thy1-induced nephritis [9,20,21,22,23]. Similar to these data, everolimus was also reported to inhibit the proliferation, activation and matrix expansion of mesangial cells in a reversible anti-Thy1-induced nephritis rat model [9]. In chronic anti-Thy1 nephritis, the formation of focal segmental glomerulosclerosis lesions, the degree of interstitial fibrosis and the increase in proteinuria over 14 weeks were ameliorated by everolimus treatment [24]. Increased glomerular hypertrophy observed in the vehicle-treated rats was completely prevented in the everolimus-treated nephritic rats [24]. Increased glomerular fibronectin mRNA and protein as well as the renal influx of monocytes/macrophages were significantly reduced in the everolimus group [24]. Consistent with these findings, our data suggest that daily treatment with everolimus after injection of anti-Thy1 to induced glomerulonephritis prevented the progression of renal injury and glomerular damage via a reduction in inflammation, oxidative stress and apoptosis and the prevention of the loss of glomerular nephrin as an essential protein for proper function of the glomerular endothelial barrier [25].

It is worth mentioning that everolimus treatment until the end of the proliferative phase of anti-Thy1-induced nephritis (approximately 2 weeks) caused marked destruction of the glomerular architecture leading to a 60% mortality rate [9]. Additionally, a progressive glomerulosclerosis with forms of structural damage such as hypocellular fibrotic lesions, hyalinosis, adhesions and capillary collapse in more than 20% of all glomeruli after approximately a month in surviving rats was also reported [9]. The authors suggest that these deleterious effects on capillary repair could be attributed to the downregulation of VEGF [9]. In this scenario, everolimus-induced downregulation of VEGF could convert the spontaneous and complete glomerular healing process occurring after glomerular injury in anti-Thy1-induced nephritis into a chronic progressive renal disease model with progressive apoptosis, crescent formation and glomerulosclerosis [9,14]. Everolimus also induces proteinuria and renal deterioration in the remnant kidney model in rats via the inhibition of the chronic glomerular repair process and the reduction in VEGF expression [15]. The potential deleterious effects of everolimus are further supported by clinical observation that renal injury might be associated with mTOR inhibitors and induction of autophagy by *everolimus* aggravates tubular dysfunction during recovery from kidney injury [26]. Extreme vigilance should be also used when prescribing everolimus for metastatic breast cancer because of some reports that everolimus can lead to acute renal injury. Thus, special caution regarding the acute vs. chronic use of everolimus in diseases with extensive glomerular cell injury should be taken into consideration, where at least adaption to a low dose of everolimus should be considered.

Bone marrow-derived stem cells (BMDSCs) contribute to cell turnover and repair in various tissue types, including the kidneys [27,28]. Mouse bone marrow cells have been shown to be able to differentiate into glomerular cells [29]. In a rat model of anti-Thy1-induced nephritis, differentiation of invading bone marrow cells into endothelial or mesangial cells was also previously reported [30], suggesting the regular contribution of bone marrow to glomerular cell turnover. Previous findings have shown that mesenchymal stem cells (MSCs) improve renal injury in anti-Thy1 nephritis by preventing the increase in serum creatinine, proteinuria and glomerular histopathology, and this could be mainly attributed to the reduction in cytokine-induced inflammation [4]. Kunter et al. have previously demonstrated that MSCs also reduced mesangiolysis in anti-Thy1-induced nephritis when injected into the renal artery, but not when infused i.v. In fact, treatment with MSCs via renal artery resulted in the recovery of serum creatinine to baseline levels with a significant reduction in proteinuria [16]. Furthermore, MSCs had striking effects on glomerular pathology, as half of the glomeruli were spared from any lesions [31,32]; in contrast, in non-MSC-treated rats, anti-Thy1 induced mesangioproliferative glomerulonephritis that is characterized by an initial mesangiolysis followed by reparative overgrowth of mesangial cells [31,32,33].

Although i.v. injection of stem cells could target injured areas and participate in tissue regeneration by differentiating into mature tissue cells [17,18] and/or inducing the release of growth factors [17,18,34], tracking MSCs after their i.v. injection demonstrated very few MSCs in the glomerular tuft, interstitium and renal tubules, suggesting that MSCs might not participate in glomerular repair by replacing injured glomerular cells [4]. However, the protective effect of MSCs in improving anti-Thy1-induced glomerulonephritis could be attributed to suppression of the local and systemic release of inflammatory cytokines and growth factors involved in the pathogenesis of anti-Thy1 nephritis [4]. Our data suggest that i.v. bolus administration of BMDSCs did not improve anti-Thy-1-induced glomerulonephritis and renal apoptosis, although it did reduce the expression of renal T cells, macrophages and IL-17. One limitation to the failure of BMDSCs to improve glomerulonephritis in our study is the use of only one bolus dose after one week of anti-Thy1 injection as well as using i.v. route of administration which might not be the proper way for target trafficking of BMDSCs to the kidney to promote glomerular healing. Our hypothesis is supported by the previous finding that intra-arterial as opposed to the commonly used i.v. injection of adult MSCs provided a proper tool to accelerate glomerular healing in a rat model of mesangioproliferative glomerulonephritis as evident by lowering of proteinuria, prevention of acute renal failure and improved resolution of mesangiolytic damage in MSC-treated kidneys [16].

In conclusion, although daily treatment of everolimus to anti-Thy1-injected rats reduced glomerular damage, renal oxidative stress, inflammation and apoptosis, the safety of long-term everolimus use remains questionable based on some recent clinical reports. However, our result clearly demonstrated the beneficial effect of using everolimus as an immunosuppressant drug to reduce renal inflammation and damage in anti-Thy1-induced glomerulonephritis. Contrary to everolimus, i.v. administration of a single bolus dose of BMDSCs one week after induction of glomerulonephritis failed to improve renal injury, although it lowered the expression of renal T cells and IL-17, suggesting that the reduction in immune cell infiltration might not necessarily translate to a reduction in renal injury in anti-Thy1-induced nephritis in rats. Future studies will focus on using higher doses of BMDSCs vs. repeated administration of the currently used bolus dose of BMDSCs to determine why BMDSCs failed to provide renal protection in anti-Thy1-induced glomerulonephritis. Additionally, comparative study of i.v. administration of BMDSCs vs. direct injection via renal artery will be also considered in our future experiments.

## 4. Methods

Four groups of 12–13-week-old male Sprague Dawley rats were used in this study (n = 6–8/group). All experiments were conducted in accordance with the National Institutes of Health Guide for the Care and Use of Laboratory Animals, and use was approved and monitored by the Augusta University Institutional Animal Care and Use Committee. Animals were housed under conditions of constant temperature and humidity and exposed to a 12:12 h light–dark cycle. All rats were given free access to rat chow and tap water.

Anti-Thy1 glomerulonephritis was induced in Sprague Dawley rats by a single injection of an antibody against Thy1 (anti-CD90, 1 mg/kg, i.v. in tail vein) (Antibody Solutions, Santa Clara, CA, USA). Rats were then treated with the immunosuppressant everolimus (2 mg/kg daily in drinking water) (Selleckchem, Houston,TX, USA) after anti-Thy1 injection or injected with single bolus dose of bone marrow-derived stem cells (BMDSCs, 10^6^ cells/200 µL of PBS, i.v. in tail vein) after a week of injection of anti-Thy1. A nontreated group of Sprague Dawley rats was used to serve as control. Because preliminary study demonstrated that anti-Thy1 injection in the currently used dose requires at least one week to induce a marked degree of glomerular injury, rats were placed in a metabolic cage for 24 h urine collection 9 days after injection of anti-Thy1 to be sure of an established degree of glomerular injury. Rats were then euthanized to collect blood and kidney. Glomeruli were isolated from one kidney to assess glomerular albumin permeability, and the other kidney was used for histological, immunohistochemical and flow cytometry analyses.

### 4.1. Isolation of BMDSCs

Leg bones from donor Sprague Dawley rats were harvested after being cleaned from muscle for isolation of BMDSCs as previously published [35]. Briefly, the soft tissue was removed before cutting the bones to expose the marrow cavity. The marrow was flushed out of the bones by inserting a 27 gauge needle connected to a 3 mL syringe filled with medium into the cavity and washing out the central core of cells. Cell suspension was harvested into a 15 mL tube through 70 mm nylon mesh to remove any smaller clumps of cells. The cells were spun down and pellet was resuspended in the medium before the counting of only nucleated large cells to eliminate any erythrocytes. Isolated bone marrow cells were incubated with Sca1+ antibody and sorted via the magnetic-activated cell sorting (MACS) technique as previously described [36]. Once the cells were sorted, a hemocytometer was used to count the cells, and subsequently, a solution of 5 × 10^5^ to 1 × 10^6^ cells/200 µL of PBS was prepared for injections. Injections were performed immediately i.v. in the rat tail vein.

### 4.2. Urine Analysis

Urinary albumin, nephrin and podocalyxin excretion levels were determined as indices of renal injury using ELISA kit (Ethos Biosciences, PA, USA). Urinary thiobarbituric acid reactive substances (TBARs, Cayman Chemical, MI, USA) excretion level was assessed as a marker of oxidative stress. Urinary and plasma creatinine levels were assessed using picric acid method and used to calculate creatinine clearance. Urinary monocyte chemotactic protein-1 (MCP-1, BD Biosciences, MA, USA) excretion level was measured as an index of inflammation.

### 4.3. Assessment of Glomerular Albumin Permeability

Kidney was placed in PBS containing phenylmethylsulfonylfluoride (1 mM) to isolate glomeruli by a gradual sieving technique as we previously described [37]. Glomerular albumin permeability was determined in isolated glomeruli as previously described [37]. Briefly, images of 10 glomeruli suspended in 5% bovine serum albumin (BSA) were captured using an inverted microscope before and after a medium change to 1% BSA.

### 4.4. Renal Histopathology

Kidney sections from different rat groups (n = 4 rats/group) were stained with hematoxylin and eosin (H&E) to assess glomerular injury and any histopathological changes in a blind fashion. Ten microscopic images of the kidney section per rat were randomly taken at 200× magnification. For immunohistochemical detection of nephrin expression, kidney sections were fixed using paraformaldehyde, washed with PBS and incubated for 1 h with normal goat serum in PBS. Slides were then incubated overnight at 4 °C with goat anti-nephrin (Santa Cruz Biotechnology, CA, USA) primary antibodies. On the second day, washing and blocking procedures were repeated before slides were incubated for 1 h with anti-goat secondary antibodies (Molecular Probes, CA, USA). Slides were then examined by confocal microscopy (LSM 510, Carl Zeiss) at 200× magnification. Images were collected from five grids from each slide.

### 4.5. Terminal dUTP Nick End-Labeling Analysis (TUNEL)

TUNEL analysis was performed using the ApopTag Fluorescein In Situ Apoptosis Detection Kit (Millipore, Temecula, CA, USA) according to the manufacturer’s specifications. Briefly, kidney sections from each rat group were fixed using paraformaldehyde and ethanol–acetic acid solution (2:1). Sections were incubated with terminal deoxynucleotidyl transferase followed by incubation with anti-digoxigenin conjugate. Propidium iodide (1 µg/mL) was added as a nuclear counterstain. Coverslips were applied using Vectashield mounting medium for fluorescence (Vector Laboratories, Burlingame, CA, USA). Images were obtained using confocal microscopy (LSM 510, Carl Zeiss) with 100× magnification. Whole kidney section was scanned for positive green fluorescent cells as an indicator of apoptosis (n = 4/group).

### 4.6. Analytical Flow Cytometry

Phenotypic analysis of renal cells were performed as described previously [38]. Briefly, kidney samples were sieved through a cell strainer (BD Biosciences, San Diego, CA, USA) followed by centrifugation (1500 rpm, 10 min) to prepare single-cell suspensions. Cells were treated with fluorochrome-conjugated antibodies of interest based on manufacturers’ instructions. Antibodies against pan T cells (CD3), IL-17, macrophages (CD68) and apoptosis (caspase 3) were all purchased from BD Biosciences. Cells then run through a four-color flow cytometer (FACS Calibur, BD Biosciences, San Diego, CA, USA), and data were collected using Cell Quest software. Samples were double-stained with control IgG and cell markers to assess any spillover signal of fluorochromes. Gating was used to exclude dead cells and debris using forward and side scatterplots. In each analysis, 100,000 total events were collected. As a gating strategy, for each sample, isotype-matched controls were analyzed to set the appropriate gates. For each marker, samples were analyzed in duplicate measurements. To minimize false-positive events, the number of double-positive events detected with the isotype controls was subtracted from the number of double-positive cells stained with corresponding antibodies (not isotype control), respectively. Cells expressing a specific marker were reported as a percentage of the number of gated events.

### 4.7. Statistical Analysis

All data are presented as mean ± SEM. Data were analyzed using one-way analysis of variance (ANOVA) followed by Tukey’s post hoc test for multiple group comparisons. Differences were considered statistically significant with *p* < 0.05 compared to the control. Analyses were performed using Graph Pad Prism Version 4.0 software.

## 5. Conclusions

The potential use of everolimus in a PPPM approach is highly appealing. PPPM is an innovative and relatively new approach in healthcare. It employs the capacity to predict disease development via not only understanding its molecular mechanism but also mitigating the adversarial effects [39]. The two key points of the PPPM are the detection of subclinical abnormalities and a drug-based correction of these abnormalities when detected [40]. Everolimus could be used as an effective pharmacologic modality in the treatment of glomerulonephritis in the early stages, which could be considered an example of a PPPM in an actual pathologic paradigm [41]. Thus, in addition to being an invaluable clinical approach, PPPM has the potential to effectively resolve certain levels of discrepancies regarding therapeutic modality, specifically about everolimus and kidney injury investigated in our study. Personalization of medicine not only improves the patient outcomes, but also could be a very valuable predictive and prognostic tool during glomerulonephritis and kidney injury in general. While warranting further research, our findings magnify the significant potential of PPPM in prevention and treatment of chronic renal injuries.
